# A comparison of blind intubation with the intubating laryngeal mask FASTRACH™ and the intubating laryngeal mask Ambu Aura-i™ a prospective randomised clinical trial

**DOI:** 10.1186/s12871-019-0718-6

**Published:** 2019-03-30

**Authors:** R. Schiewe, M. Stoeck, M. Gruenewald, J. Hoecker, B. Bein

**Affiliations:** 1 Department of Anaesthesiology and Intensive Care Medicine, Asklepios Hospital St. Georg, Lohmuehlenstr. 5, D-20099 Hamburg, Germany; 2 Department of Anaesthesiology and Intensive Care Medicine, University Hospital Schleswig-Holstein, Campus Kiel, Schwanenweg 21, D-24105 Kiel, Germany

**Keywords:** Laryngeal mask airway, Laryngeal mask guided tracheal intubation, Airway management, Fiberoptic intubation

## Abstract

**Background:**

The intubating laryngeal mask Fastrach™ is considered a gold standard for blind intubation as well as for fibreoptic guided intubation via a laryngeal mask. Recently, a single use version of the mask has been introduced. We compared the Fastrach single use with the new, low-priced single use intubating laryngeal mask Ambu Aura-i™.

We hypothesised that the LMA Ambu Aura-i and the LMA Fastrach are comparable with respect to success rates for mask placement and blind tracheal intubation through the LMA device.

**Methods:**

A prospective, randomised clinical trial. University Hospital Schleswig-Holstein, Campus Kiel, from April 2011 to April 2012.

Eighty patients undergoing general anaesthesia with planned tracheal intubation were randomised and enrolled in the study. Blind intubation was performed with either laryngeal mask using two different tracheal tubes (Rüsch Super Safety Silk™ and LMA ETT™). A crossover-design was performed after an unsuccessful procedure.

Primary outcome measure was the overall success rate of blind intubation. Secondary outcome measures were the time to the first adequate ventilation, a subjective handling score, and a fibreoptic control of placement, as well as the success rate of mask placement, time for mask removal after successful intubation, differences in airway leak pressure, and the incidence of postoperative sore throat and hoarseness.

**Results:**

The success rate of tracheal intubation with the Fastrach for the first and second attempt was significantly better compared with the Ambu Aura-i. Tracheal intubation was also significantly faster (14.1 s. ±4.4 versus 21.3 s. ±9.0; *p* < 0.01), and the time interval for mask removal after successful intubation was significantly shorter using the Fastrach device (24.0 s. ±8.2 versus 29.4 s. ±7.5; *p* < 0.001). There were no significant differences between groups regarding the incidence of postoperative sore throat and hoarseness.

**Conclusion:**

Both laryngeal mask devices are suitable for ventilation and oxygenation. Blind intubation remains the domain of the LMA Fastrach, the Ambu Aura-i is not suitable for blind intubation.

**Trial registration:**

Clinicaltrials.gov Identification Number NCT03109678, retrospectively registered on April 12, 2017.

## Background

After development of laryngeal masks (LMA) in the 1980s, there have been anecdotical reports on their use in difficult airway management and during “cannot intubate, cannot ventilate” situations. In 1997, the intubating laryngeal mask Fastrach™ was introduced [1]. LMA Fastrach™ was developed for placing tracheal tubes without fibreoptic assistance [2]. With reported rates for successful intubation of 75% on the first attempt, and 99,7% with fibreoptic assistance [3, 4], LMA Fastrach became the reference for LMA assisted tracheal intubation. Following the increasing demand for single use equipment due to cleaning and sterilisation issues, a single use Fastrach was developed and introduced into the market. However, high costs hamper a more widespread use in clinical routine. Recently, similar and lower-priced intubating LMAs have been developed, such as the LMA Ambu Aura-i™. With this study, we investigated the feasibility of blind intubation using the LMA Ambu Aura-i and compared it with the LMA Fastrach as contemporary reference.

We hypothesised that the LMA Ambu Aura-i and the LMA Fastrach are comparable with respect to success rates for mask placement and tracheal intubation. Also, we supposed that tracheal intubation would be more successful in both laryngeal masks using the endotracheal tube (ETT) specifically designed for use with the Fastrach.

## Methods

After local ethics committee approval (Ethics committee of the Christian-Albrechts-University at Kiel, Chair: Prof. Dr. H.M. Mehdorn, study Ref. No. AZ 107/02, 26.04.2011) and written, informed consent, 80 patients undergoing general anaesthesia with planned tracheal intubation for elective surgical procedures were enrolled in the study, starting on 26.04.2011. Patients were randomised to the Ambu Aura-i group (*n* = 40), and the LMA Fastrach group (*n* = 40), respectively, using a sealed envelope which had been prepared after a randomisation procedure using the website Randomization.com (http://www.randomization.com). Further, each group was divided in two subgroups to investigate the influence of different tracheal tubes on the success of LMA assisted tracheal intubation. Either a Rüsch Super Safety Silk™ (ID 7,5 mm, Rüsch, Kernen, Germany) representing a standard PVC tracheal tube, or a LMA ETT™ (ID 7,5 mm, LMA, Bonn, Germany) as a tube specifically developed for the LMA Fastrach, were used. After intubation failure, a crossover-design was performed, using the other LMA or the other tracheal tube.

Primary endpoint was the overall success rate of blind intubation with either mask after maximum of two attempts. Secondary endpoints were the influence of the tracheal tubes, equivalence of the masks regarding fibreoptic visualisation, a subjective handling score, differences in airway leak pressure, and the incidence of postoperative sore throat and hoarseness.

Inclusion criteria were the presence of all of the following: general anaesthesia with planned tracheal intubation, elective surgery, written, informed consent.

Exclusion criteria were the presence of at least one of the following: ASA physical status IV and V, severe pulmonary comorbidity (COPD GOLD >III, bronchial asthma), indication for rapid-sequence induction, mouth opening (interincisor distance) < 3 cm, and morbid obesity (BMI > 35 kg.m^− 2^).

The study investigators were three anaesthesiologists very well experienced in using different kinds of laryngeal mask devices, including both LMA devices compared in this study (BB, MS, JH).

All patients enrolled in the study were pre-medicated with midazolam 7.5 mg p.o. 30 min before the procedure with a sip of water. Routine monitoring included 5 lead ECG, SpO_2_ and heart rate, as well as non-invasive blood pressure measurement. Depth of anaesthesia was monitored with bispectral index (BIS 2000 XP™, Aspect Medical Systems, Wallingford, USA), neuromuscular monitoring was performed by relaxometry (GE Healthcare, Helsinki, Finland). Clinical predictors of difficult airway, such as Mallampati score, mouth opening, and thyromental distance, were recorded.

Patient’s head was placed in a neutral position. Pre-oxygenation with oxygen 100% via face mask for 3 min. Was followed by a standardised induction of anaesthesia using propofol 2 mg.kg^− 1^ lean body weight and remifentanil 0.3 μg.kg^− 1^.min^− 1^ lean body weight. Neuromuscular blockade was achieved with rocuronium 0.6 mg.kg^− 1^ ideal body weight and anaesthesia was maintained by propofol bolus and remifentanil infusion at 0.2 μg.kg^− 1^.min^− 1^. After induction of anaesthesia, the study was started by placing the laryngeal mask (appropriate size #4 or #5, depending on patients’ body weight) into the hypopharynx when an adequate depth of anaesthesia was recorded (BIS between 40 and 60). Cuffs were inflated according to the manufacturers’ instructions (30/40 ml air), and time (T1) was recorded between picking up the laryngeal mask and the first successful ventilation. Successful ventilation was defined as positive capnometry combined with thorax excursions. When the first attempt at mask placing failed, a second attempt was allowed, and total time was then documented as T1. After a failure with the second attempt, a crossover-design with the alternate device was performed.

A subjective handling score was recorded, graded in “excellent”, “good”, “fair”, and “poor”. Laryngeal mask airway leak pressure (cm H_2_O) was recorded by setting the APL valve to 40 cm H_2_O, and fresh gas flow at 3 l/min. The presence of audible leakage as well as the absence of corresponding pressure increase on the monitor was documented as leakage. A stethoscope was used to distinguish between oral or gastric leakage.

Next, a fibreoptic evaluation of LMA placement was performed. With a fibrescope (Karl Storz, Tuttlingen, Germany), the position of the larynx relative to the laryngeal cuff and mask-aperture was visualised and categorised as “correct”, “lateral deviation”, “epiglottic downfolding” or “not assessable”. Additionally, the view on the larynx comparable to Cormack/Lehane score was recorded. After relaxometry detected a TOF ratio of 0, the tracheal tube was placed through the respective LMA without any optical assistance. Time (T2) was stopped from picking up the tracheal tube until the first successful ventilation. If the first attempt was unsuccessful, an immediate second attempt with optimised LMA positioning and patient’s head reclination was performed, and the total time (T2a) was recorded. If also the second attempt of tracheal tube placement was unsuccessful, a crossover-design with the alternate tracheal tube was performed identical to the attempt with the first tracheal tube, yielding time T2b. If the alternate tracheal tube could also not be placed correctly, the attending anaesthesiologist placed the tube with fibreoptic assisted (time T2c). The attempt was terminated and the attempt classified as “failure” if total time exceeded 300 s or SpO_2_ decreased to < 91%.

After successful intubation, time was stopped for mask removal over the tracheal tube, either using the removal bar developed specifically for the LMA ETT™ or with a conventional Magill forceps for the Rüsch tube. Time (T3) was recorded from removal of the tube connector until successful ventilation. Finally, tracheal tube position was evaluated by bilateral auscultation using a stethoscope. Unilateral ventilation or accidental extubation upon LMA removal were also recorded.

On the day after surgery patients were interviewed by an investigator blinded to group assignment, and the incidence and extent (none/moderate/severe) of postoperative sore throat and hoarseness were recorded.

Statistical analyses were performed using Graph Pad version 6.00 for Windows (GraphPad Software, La Jolla California USA, www.graphpad.com). Sample size was calculated using Stat Mate version 2.00 for Windows (GraphPad Software, La Jolla California USA, www.graphpad.com). An estimated success rate for blind intubation of 60% in the Aura-i group versus 90% in the Fastrach group yielded a sample size of *n* = 38 for α = 0.05 and β = 0.20. To compensate for dropouts, *n* = 40 subjects were enrolled in each group. Data were analysed regarding normal distribution by D’Agostino and Pearson Test (omnibus normality test). Normally distributed data were analysed by one-way-ANOVA, followed by Bonferroni correction for multiple comparisons if appropriate. Non-normal data were analysed by Kruskal-Wallis test. Proportions were compared with Fisher’s exact test or the Chi-square test, as appropriate. Study data are presented as mean (SD) or median (IQR).

The study was retrospectively registered on Clinicaltrials.gov, Identification Number NCT03109678.

## Results

**Fig. 1:**
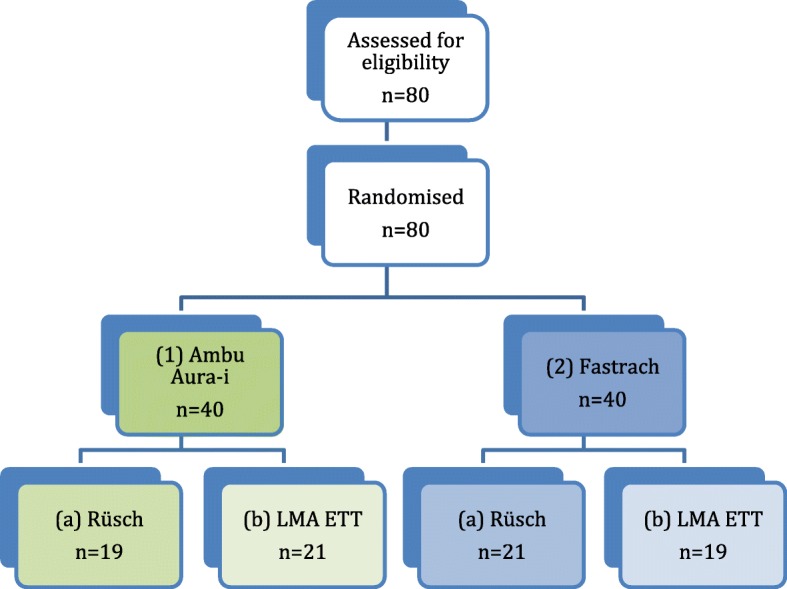
*Flow chart study design.* 80 patients randomised; two laryngeal mask device groups (Ambu Aura-i™ and Fastrach™) with two subgroups each using two different tracheal tubes (Rüsch Super Safety Silk™ and LMA ETT™)

Eighty patients were enrolled in the study, and all patients were analysed (Fig. 1). There were no significant differences between groups with regard to demographic data, ASA physical status (Table 1), or clinical predictors of difficult airway, such as Mallampati score, mouth opening, and thyromental distance (Table 2)

**Table 1:** Demographic data and ASA status

Combination LMA/tube	Subgroup 1a)	Subgroup 1b)	Subgroup 2a)	Subgroup 2b)	Total
	Aura-i/Rüsch	Aura-i/ETT	Fastrach/Rüsch	Fastrach/ETT	
Number	*n* = 19	*n* = 21	*n* = 21	*n* = 19	*n* = 80
Height [cm]	170 ± 12.9	172 ± 10.9	175 ± 10.8	169 ± 13.5	172 ± 12.0
	[147–200]	[152–198]	[160–197]	[154–200]	[147–200]
Weight [kg]	80 ± 18,5	76 ± 13.3	76 ± 12.1	70 ± 14.6	75 ± 14.8
	[47–105]	[57–105]	[58–100]	[46–110]	[46–110]
Age [years]	59 ± 15.1	58 ± 16.9	60 ± 12.6	57 ± 19	58 ± 15.7
	[32–90]	[25–83]	[37–82]	[15–81]	[15–90]
Sex [w:m (%)	8: 11	10: 11	7: 14	12: 7	37: 43
	(42: 58)	(47: 53)	(33: 67)	(63: 37)	(46: 54)
ASA I/II/III (%)	2/12/5	5/12/4	4/15/2	3/10/6	14/49/17
	(10/64/26)	(24/57/19)	(19/71/10)	(16/52/32)	(17/61/21)

Values are median ± SD [minimum – maximum] or number (percent). No significant differences

**Table 2:** Clinical predictors of difficult airway

Combination LMA/tube	Subgroup 1a)	Subgroup 1b)	Subgroup 2a)	Subgroup 2b)	Total
	Aura-i/Rüsch	Aura-i/ETT	Fastrach/Rüsch	Fastrach/ETT	
Mallampati I/II/III	7/9/3	11/8/2	13/7/1	10/5/4	41/29/10
	(37%/47%/16%)	(52%/38%/10%)	(62%/33%/5%)	(53%/26%/21%)	(51%/36%/13%)
Interincisor distance [cm]	4.3 ± 0.7	4.2 ± 0.5	4.4 ± 0.7	4.3 ± 0.7	4.3 ± 0.6
	[3.5–6.2]	[3.2–5.5]	[3.2–6.0]	[3.5–5.8]	[3.2–6.2]
Thyromental dist. (Patil) [cm]	7.8 ± 1.9	7.5 ± 1.1	8.2 ± 1.1	7.6 ± 1.5	7.8 ± 1.4
	[4–11]	[4.8–10]	[5.5–10]	[4.5–11]	[4–11]

Values are absolute values (percent) and mean ± SD [minimum – maximum]. No significant differences

LMA placement on the first attempt was successful in 87.5% (Ambu Aura-i 82.5%, Fastrach 92.5%), and in all patients (100%) on the second attempt. Therefore, a second attempt was required in 17.5% of the Ambu Aura-i, and in 7.5% of the Fastrach group (*p* > 0.05). No patient had to be assigned to the alternate device.

**Fig. 2:**
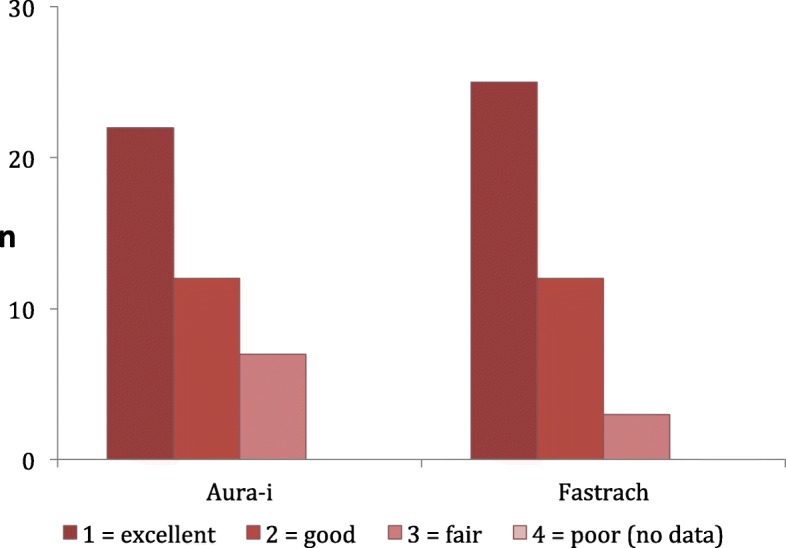
*Subjective handling score of the two LMA devices.* n = number, handling rated as excellent, good, fair, or poor

There was a significant difference regarding the time to the first successful ventilation (T1) between both groups. The LMA Fastrach could be placed after 15.9 s (SD ± 7.0) on the first attempt, the LMA Ambu Aura-i after 18.8 s (SD ± 7.6) (*p* = 0.017). Mask size (#4 vs. #5) had no influence on time required for placement. There was no significant difference regarding the subjective handling score. Both masks were rated either “excellent” or “good” (Fig. 2).

**Fig. 3:**
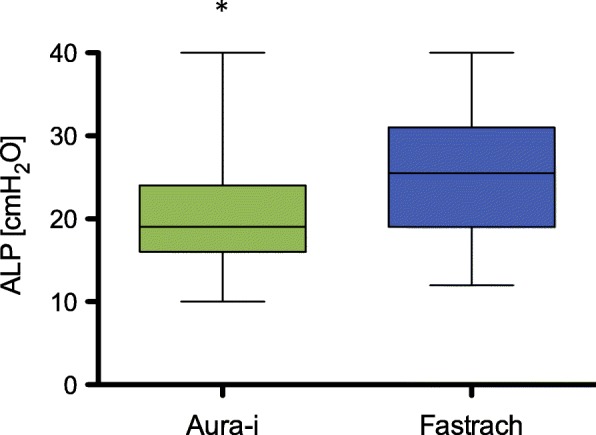
*Airway leak pressure in both LMA device groups.* Box-Whisker-Plot, showing Mean, IQR, Minimum, Maximum, * *p* < 0.001

Airway leak pressure was significantly lower (*p* < 0.001) in the Ambu Aura-i group (mean 19cmH_2_O, SD ±6) compared to the LMA Fastrach group (mean 26cmH_2_O, SD ±8), (Fig. 3).

Regarding the primary endpoint of the study, there was a significant difference between both LMA groups regarding successful blind intubation with the first tracheal tube:

Group Aura-i-Rüsch 9 (47%)/Fastrach-Rüsch 17 (81%), *p* < 0.05; group Aura-i-ETT 4 (81%)/Fastrach-Rüsch 17 (81%), *p* < 0.01; group Aura-i-Rüsch 9 (47%)/Fastrach-ETT 17 (90%), *p* < 0.01; group Aura-i-ETT 4 (19%)/Fastrach-ETT 17 (90%), *p* < 0.01; group Aura-i-Rüsch 9 (47%) + Aura-i-ETT 4 (19%)/Fastrach-Rüsch 17 (81%) + Fastrach-ETT 17 (90%), *p* < 0.01.

There was no significant difference on success of blind intubation with the first tracheal tube within either LMA-group (group Aura-i-Rüsch 9 (47%)/Aura-i-ETT 4 (19%), *p* = 0.092; Fastrach-Rüsch 17 (81%)/Fastrach-ETT 17 (90%), *p* = 1.000).

Tracheal tubes had no significant influence on success of blind intubation (group Aura-i-Rüsch + Fastrach-Rüsch/Aura-i-ETT + Fastrach-ETT, *p* = 0.268). Regarding the crossover attempts, Table 3 clarifies the poorer performance of the LMA Aura-i.

**Table 3:** Failed attempts of blind intubation in each subgroup, including attempts of *crossover-design*

	Subgroup 1a)	Subgroup 1b)	Subgroup 2a)	Subgroup 2b)
	Aura-i/Rüsch	Aura-i/ETT	Fastrach/Rüsch	Fastrach/ETT
Number	*n* = 36	*n* = 31	*n* = 23	*n* = 23
Attempts	62	61	29	29
Failed	49	57	11	10
Failed (%)	79%	93%	38%	34%
	*, **, ***	$, §		

^*^ *p* = 0.03 compared to 1b, ^**^ *p* = 0.0003 compared to 2a, ^***^ *p* < 0.0001 compared to 2b

^$ ^*p* < 0.0001 compared to 2a, ^§^ *p* < 0.0001 compared to 2b

Values are absolute values

Fibreoptical tracheal tube placement was necessary more often in group 1 (*n* = 23) compared with group 2 (*n* = 3), *p* = 0.0005.

Using the Ambu Aura-i compared to LMA Fastrach leads to more cases of laryngeal lateralization (4/40 (10%) versus 1/40 (25%), *p* = 0.17), as well as significant more cases of epiglottic downfolding (13/40 (33%) versus 4/40 (10%), *p* < 0.02).

**Fig. 4:**
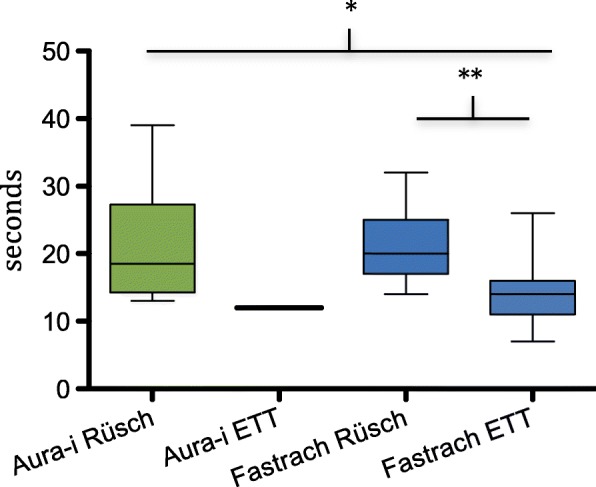
*Time for successful intubation on the first attempt in all subgroups, in seconds.* Box-Whisker-Plot, showing Mean, IQR, Minimum, Maximum, * *p* < 0.05, ** *p* < 0.01

There was a significant difference between the groups regarding the time for tracheal intubation (T2). The combination of LMA Fastrach/LMA ETT showed the best results regarding overall success rates and regarding the time for tracheal intubation (Fig. 4; group 1a/2a, *p* = 0.966; group 1a/2b, *p* < 0.05; group 2a/2b, *p* < 0.01).

Regarding the time interval for mask removal (T3), using the LMA ETT removal bar was significantly faster than using the Magill forceps with the Rüsch tube (24 s. ±7.5, versus 29.4 s. ±8.2; *p* < 0.001). No influence of the LMA device on removal time could be detected.

Tracheal tube displacement occurred only once in the Ambu Aura-i/LMA ETT subgroup.

Postoperative interviews did not reveal any significant difference between either group regarding the occurrence of patient discomfort (Table 4).

**Table 4:** Postoperative interview about the intensity (none, moderate, severe) of different types of patient discomfort (sore throat, difficulty swallowing, hoarseness) in each subgroup

	Sore throat				Difficulty swallowing				Hoarseness			
Subgroup	1a	1b	2a	2b	1a	1b	2a	2b	1a	1b	2a	2b
None	14	14	15	16	15	17	17	16	15	16	16	16
%	74%	67%	71%	84%	79%	81%	81%	84%	79%	76%	76%	84%
Moderate	4	5	4	3	1	3	1	3	3	4	2	3
%	21%	24%	19%	16%	5%	14%	5%	14%	16%	19%	10%	16%
Severe	0	1	0	0	2	0	1	0	0	0	1	0
%	0%	5%	0%	0%	11%	0%	5%	0%	0%	0%	5%	0%

Values are absolute values and percent. No significant differences

## Discussion

Introducing the intubating laryngeal mask Fastrach in 1997 was a milestone in modern airway management. While LMAs had been anecdotically used for difficult airway situations so far, now a secure airway could be easily established over the laryngeal mask airway [5]. Blind intubation procedures using the intubating LMA could be performed with success rates of more than 90% [1]. A fibreoptic guidance of the tracheal tube was usually not necessary for a successful intubation [3]. Subsequent studies confirmed these results, and the LMA Fastrach was considered as “gold standard” for laryngeal mask guided intubation. Some authors even suggested LMA guided intubation not only as a backup procedure, but also for primary usage in specific patient populations [6], as well as an alternative in preclinical emergency medicine [7]. Despite the benefits of intubating laryngeal masks, there were also limitations and disadvantages. Ulcerations of the laryngeal mucous membrane [8] were reported, and the LMA ETT was not suitable for long-term mechanical ventilation due to its cuff design [9]. Also, relatively high acquisition costs of the LMA Fastrach device and its corresponding specific tracheal tube encouraged the search for alternatives. However, their quality and performance in comparison to the existing standard has to be evaluated carefully [10].

Primary endpoint of our study was the overall success rate of blind intubation with either mask within two attempts. While high success rates regarding the LMA Fastrach had been published before [11, 12].

A recent study compared tracheal intubation using the Ambu Aura-i and a flexible intubating scope with blind intubation using LMA Fastrach. The data suggest that intubation with the LMA Fastrach is faster but that first-attempt and overall intubation success rates were comparable in both groups [13].

There were no data for the Ambu Aura-i mask regarding blind tracheal intubation available so far.

Our data confirm the efficiency of the LMA Fastrach.

Blind intubation success rates were significantly lower using the Ambu Aura-i mask, both regarding overall success rates as well as regarding the success rates in the predefined subgroups. Using the Ambu Aura-i with the Rüsch Super Safety Silk showed a success rate of 42% with the first attempt, while the success rate decreased to only 5% with the LMA ETT. An optimised LMA position was not able to improve these results.

The Rüsch tube performed better with respect to overall success rates. Nevertheless, blind intubation with the Ambu Aura-i mask was successful in only a third of the patients, and is therefore not recommended for clinical use.

We suspect the less curvature of the Ambu Aura-i compared to the LMA Fastrach as well as the lack of tube guidance through the Ambu Aura-i as the two main reasons for the less success rate in blind tracheal intubation with this device.

For more than 50% of the patients (*n* = 23/40) in the Ambu Aura-i group, a fibreoptic assisted intubation was necessary. Overall success rate for intubation with the Ambu Aura-i mask was approximately 90%.

Even though there was a significant difference regarding fibreoptic control after placing the masks, with a better fit of the LMA Fastrach, this evaluation is limited due to different mask designs.

Regarding the fibreoptic control of LMA placement and differing descriptions in the literature [14], there is yet no definitive consensus regarding the evaluation of the mask position with the fibrescope. Our data should be interpreted with caution, because we changed the fiberscope device during the study (after a few measured patients in both LMA groups), using a small diagnostic one at first and switching to a larger therapeutic one later to get a better lightning and a better overview. Nevertheless, using the Ambu Aura-i compared to LMA Fastrach leads to more cases of laryngeal lateralization, as well as epiglottic downfolding. Though these differences did not reach statistical significance in our study, they could be meaningful in individual subjects.

Both laryngeal mask devices could be placed at least with the second attempt, and therefore qualify for clinical use. Even though in our study the LMA Fastrach could be placed significantly faster than the Ambu Aura-i (16 s. versus 19 s.), we consider this time difference as negligible in a clinical context. Our results confirm previously reported ranges [15]. Furthermore, both devices were rated “excellent” or “good” by the investigators.

Airway leak pressure (ALP) is a commonly used method to quantify the efficiency of the airway seal [16]. In our study, we inflated cuffs with manufacturer recommended volumes to get comparable data. Furthermore, our study should not detect the smallest possible cuff volume, but the highest possible ALP. In a study with the LMA Unique, ALP was highest when the cuff was completely inflated [17]. Our analysis of the ALP showed significant differences between both devices (Ambu Aura-i 19 cmH_2_O versus LMA Fastrach 26 cmH_2_O) in favour of the LMA Fastrach, confirming existing data with ALP ranging from 25 to 30 cmH_2_O [18]. Our study did not investigate the possibility of adverse events caused by higher ALP values. However, the LMA Fastrach group presented more cases of gastric insufflation (12/40 versus 7/39), possibly due to the ALP being higher than the lower oesophageal sphincter tone. Neither mask provides a drainage channel for gastric tube placement [19].

Regarding laryngeal mask removal over the tracheal tube, we compared two different procedures depending on which laryngeal mask was used: either using the removal bar specifically designed for the LMA ETT or a conventional Magill forceps for the Rüsch tube. Recorded times were significantly shorter using the LMA ETT system (24 s. ±8 versus 29 s. ±7). In our opinion, this difference is not due to the laryngeal mask used, but rather due to the large pilot-cuff of the Rüsch tube that needs to be fully deflated to pass it through the laryngeal mask.

Concerning airway discomfort after the procedure, about one third of the patients reported discomfort, but most of the patients were devoid of any symptoms. There were no significant differences between both groups, regarding the different types of discomfort, even though there were more (unsuccessful) attempts of intubation in the Ambu Aura-i group.

Our study has some limitations. (1) There were no patients with a difficult airway in each group. (2) The design of the study does not allow any statement about the equivalence of LMA guided placement compared to primary fibreoptic guided intubation (3) The fibreoptic evaluation of the laryngeal view analogous to the Cormack/Lehane score was not diagnostically conclusive.

## Conclusion

In conclusion, the results of our study suggest that both laryngeal mask devices are suitable for ventilation and oxygenation. Blind intubation using an intubating LMA remains the domain of the LMA Fastrach device because of its high success rates. The success rate does not differ with respect to the tube used. The Ambu Aura-i device is not suitable for blind intubation. Tracheal intubation using this device should be performed with fibreoptic assistance, as recommended by the manufacturer.

## Abbreviations

ALP: Airway leak pressure
APL: Valve adjustable pressure-limiting valve
BIS: Bispectral index
BMI: Body mass index
COPD: Chronic obstructive pulmonary disease
ETT: Endotracheal tube
LMA: Laryngeal mask
